# Gender Differences in the Relationship between Interpersonal Trust and Innovative Behavior: The Mediating Effects of Affective Organizational Commitment and Knowledge-Sharing

**DOI:** 10.3390/bs12050145

**Published:** 2022-05-14

**Authors:** Hao Yuan, Dan Ma

**Affiliations:** 1 School of Sociology and Political Science, Shanghai University, Shanghai 200444, China; 2 Department of Sociology, Shanghai Administration Institute, Shanghai 200233, China; madansoc@163.com

**Keywords:** gender difference, interpersonal trust, innovative behavior, knowledge-sharing, affective organizational commitment

## Abstract

The innovative behavior of employees is the micro-foundation of enterprise innovation. The objective of this study was to assess the role of gender differences in the effect of interpersonal trust on employee innovation and the mediating roles of organizational commitment and knowledge-sharing. This study tested research hypotheses with a multi-group structural equation model, using data collected from 688 participants in Shanghai, China. The results showed that interpersonal trust had significant impacts on affective organizational commitment, knowledge-sharing and innovation behavior. Affective organizational commitment and knowledge-sharing mediated the effect of interpersonal trust on employee innovation. Furthermore, the direct impact of interpersonal trust on innovative behavior was significantly higher for women than for men, whereas males’ affective organizational commitment increased their knowledge-sharing behaviors. In addition, there were no significant gender differences in the effect of interpersonal trust on organizational commitment and in the effect of knowledge-sharing on innovative behavior. These results confirmed that interpersonal trust was more important for female knowledge-sharing and innovative behavior, and affective organizational commitment was more important for male knowledge-sharing.

## 1. Introduction

Employees’ innovation behavior (IB) is crucial for organizations to be competitive. It typically includes several types of behaviors, such as the creation, promotion and implementation of novel ideas in the workplace [1]. In particular, considering the complexity and ever-changing market, information and communication technological (ICT) companies have become increasingly reliant on individual-level innovative behaviors [2]. Recently, extensive research on innovation management has revealed that the organizational environment, such as the culture, social capital, team diversity and some other structural factors in the workplace, can significantly promote employee innovation [3]. Interpersonal trust in the workplace (ITW), defined as the confidence that the other party will act in an ethical and predictable manner, has led to greater organizational commitments, knowledge-sharing (KS) and more IBs [4]. Prior research has also revealed that both affective organizational commitment (AOC) and KS can be mediators between ITW and IB [5].

Female employees remain significantly underrepresented in the ICT industry. There are differences between the genders in professional research directions and career choices, which often result in males occupying dominant positions in science and technology innovation [6]. In the ICT industry, females constitute only 33% of the workforce at the entry level and 17% at the leadership level [7].

However, the innovation of ICT companies requires the cooperation of male and female employees. Gender diversity in companies can promote the employment of more skilled employees and result in greater overall success [8]. The increase in gender diversity in ICT companies has promoted stronger creativity and innovation. Gender diversity may play a central role in determining the capacity for organizational innovation as there are striking differences between the innovation-friendly pattern and the traditional gender-based stereotypes.

Nevertheless, there has only been limited progress in understanding the gender differences and the social determinants of employee innovation. Therefore, in this study, we explored the gender differences in the relationship between ITW and IB, using data from a sample of 688 respondents at 40 ICT companies in Shanghai, China. This article is articulated as follows. Section 2 reviews the conceptual framework on the gender differences and the relationships of IB with ITW, AOC and KS. Then, we propose ten hypotheses. Section 3 presents the methodology and data of the empirical research. In Section 4, we present and discuss the results from multi-level structural equation models. The final section summarizes the major findings of the study and provides our conclusion.

## 2. Literature Review

IB refers to employees’ discovery and generation of new ideas and the proposal and implementation of solutions [1,3]. It usually depends on the re-integration and absorption of knowledge, on the collision between different ideas, and on the re-integration of resources. Organization environments, such as interpersonal trust between organizational members, are influential for both KS and IB [5]. KS highly depends on the mutual trust between organizational members. Employees hardly share knowledge and information with others if they distrust each other.

ITW is derived from employees’ positive expectations that the others in the organizations will perform particular actions important to them under risk-prone conditions [9]. In most cases, employees do not have enough knowledge and experiences to implement innovations by themselves. IB is based on the sharing of knowledge, technology, information and experience. KS in ICT companies, especially those who are creating complex and knowledge-intensive products, is essential for maintaining high levels of organizational innovation [10]. In general, previous studies have confirmed the positive relationship between KS and IB. Therefore, KS is an important mediator between ITW and IB [11].

AOC is crucial to KS and employee innovation. AOC constitutes a necessary condition for employees to voluntarily share important knowledge such as competences, key information and personal experiences [12]. AOC produces a collective sense of identity among individuals in the organization, leads to a sense of shared purpose, and increases the likelihood that employees will engage voluntarily in KS within the organizations [13].

ITW includes vertical and horizontal trusts [14]. Vertical trust lies between employees and their immediate supervisors or top management, whereas horizontal trust exists between employees and their peers or equals in the workplace [15]. However, small or mid-sized ICT companies have adopted flexible, increasingly flat organizational forms with fewer hierarchical levels in order to encourage equal interactions and free communications. This makes horizontal trust, rather than vertical trust, more important for teamwork and organizational innovation [16]. Therefore, this study focused on horizontal trust.

IB is the process of reengineering knowledge and reusing resources. Interpersonal trust is conducive to resolving the conflicts among members of an innovation team and shaping organizational commitment. Stronger interpersonal trust can lead to more mutual supports, cooperation and coordination among employees and less opportunistic behaviors [17]. ITW can also provide psychological support for employees and help them obtain feedback on work-related issues, which has a positive impact on IB [18]. ITW also improves the feeling of emotional safety among the team, which can encourage IB. High interpersonal trust can help employees overcome the pressure at work and obtain encouragement for IB [9]. Therefore, we developed the following hypothesis:

**Hypothesis** **1** **(H1).** *ITW could have a positive impact on IB*.

Organizational commitment is an emotional state maintained between an employee and an organization, usually measured by three categories: affective commitment, continuance commitment and normative commitment [19]. Among these three categories, AOC has drawn the most research attention [20]. AOC refers to the emotional attachment and identification with organizational values and organizational goals [21]. ITW is derived from personal informal relationships, affective connections and high-quality interactions and leads to stronger AOC [22]. As ICT companies have less formality and consistently experienced rapid restructuring and renewal, employees are more likely to have greater interpersonal trust which has become an increasingly important element of organizational environments in uncertain times [23]. Employees usually have higher levels of AOC when they trust each other in the workplace. Therefore, we developed the following hypothesis:

**Hypothesis** **2** **(H2).** *ITW could have a positive impact on AOC*.

Another potential consequence of ITW is KS. KS requires the willingness of individuals to actively participate in the exchange and creation of knowledge and to collaborate together in the workplace [13]. KS is specifically important for ICT companies to obtain sustainable competitive advantages. These companies should encourage their employees to transfer expertise, information and technology to their colleagues [24]. KS is the fundamental means to enrich organizational knowledge by leveraging individual knowledge. Trust relationships, common frames of reference and shared goals are important antecedents of KS [25]. As knowledge, especially tacit knowledge, is a resource developed in an individual mind, KS requires a willingness of the individuals who possess it to share and communicate it [26].

However, promoting KS is a major challenge, as many employees tend to hoard their own knowledge, rather than taking steps to convert their knowledge into a form that can be understood, absorbed, and used by others, and they are suspicious of the knowledge given by their colleagues [27]. In an organization, the exchange of knowledge, technology, information and experience not only depends on formal organizational communication, such as document exchange, training, meetings and so on, but also on informal in-person communications, especially for the dissemination of tacit knowledge [28]. It is nearly impossible for employees to share ideas and knowledge without strong interpersonal trust. High levels of ITW create conditions for the flow and sharing of ideas and knowledge in organizations. Therefore, interpersonal interactions constitute the channel of information flow, reducing the time and cost of obtaining information [29]. The dissemination of knowledge, information and technology depends on interpersonal activities. Interpersonal trust can promote communications among employees, reduce the cost of knowledge and information, and lessen resource mismatch caused by information asymmetry [30].

Why is ITW so important in determining KS? There are several reasons. First, when there are trust relationships, employees will be less concerned about the loss of KS and be more likely to provide useful knowledge [31]. Employees believe that trusted colleagues, as the recipients of knowledge, will use the shared knowledge appropriately and not use it against them, even if the knowledge is incomplete, imperfect or containing errors. Second, employees expect reciprocity and receive positive feedback when in trust relationships, according to social exchange and social capital theories [9]. Third, the knowledge shared by a trusted colleague or supervisor can save time and efforts in terms of verifying the acquired knowledge. Finally, ITW encourages employees to accept others’ knowledge and improve their responsiveness in KS [32]. ICT companies can encourage more sharing behaviors with a set of normative documents that promote their duties or obligations to share knowledge in accordance with organizational values [33]. ICT companies can reduce concerns regarding the potential losses of valuable information and promote the competitive advantages resulting from knowledge diffusion [32]. Therefore, we developed the following hypothesis:

**Hypothesis** **3** **(H3).** *ITW could have a positive impact on KS*.

KS depends on the giver’s willingness to voluntarily share knowledge. A high level of AOC will result in voluntary cooperation, including the sharing of knowledge. Extensive research has confirmed that AOC is a significant determinant of KS, across a broad range of countries and occupations [34]. AOC plays an important mediating effect between ITW and KS. ICT companies depend on their highly educated talents and significantly invest in employee training and team building. The nature of knowledge in ICT companies is often tacit and less formalized. Higher AOC can encourage employees in ICT companies to voluntarily help their co-workers and share their tacit knowledge [35]. Employees’ tacit knowledge does not transform automatically or easily into organizational knowledge, even with the implementation of knowledge repositories [36]. Therefore, we developed the following hypothesis:

**Hypothesis** **4** **(H4).** *AOC could have a positive impact on KS*.

Similarly, AOC can also increase the effort and value coherence and the attachment to the organization, thus promoting IB [37]. IB is different from habitual behaviors embedded in bureaucratic systems. IB is potentially costly to employees due to the inherent risks. The failure of innovation can result in negative consequences [38]. Therefore, employees might participate in innovational activities only when they have strong AOCs and are willing to take such risks. Therefore, we developed the following hypothesis:

**Hypothesis** **5** **(H5).** *AOC could have a positive impact on IB*.

KS is part of knowledge management and has been a focus of both academia and business due to its relevance for innovative capabilities [8]. KS not only includes sending and receiving knowledge, but also enables the acquisition of beneficial knowledge [39]. KS is a crucial process for an organization to meet challenges, to gain competitive advantage and to achieve its targets in an efficient and effective way. It enables the knowledge held by individuals and groups to be transferred to the organizational level, facilitating the development of new products, services, and processes in the organizations [22]. KS is a fundamental tool for fostering innovative behaviors, stimulating critical thinking, and translating ideas into innovative behaviors [40]. Employees can voluntarily share their valuable implicit or explicit knowledge with team members or colleagues. These behaviors in the workplace lead to knowledge creation and drive organizational innovation in ICT companies [41]. Employees in ICT companies need explicit and implicit knowledge to improve their innovative performance. These companies should motivate employees to elaborate, integrate, and translate knowledge and information rather than simply passing them on to recipients [42]. Therefore, we developed the following hypothesis:

**Hypothesis** **6** **(H6).** *KS could have a positive impact on IB*.

Although a number of studies have applied a gender-focused lens to innovation research in the last decade, the gender dynamics impacting employee innovation have not been fully discussed [43]. Employee innovations in traditionally male-dominated industries have tended to exclude female participants, although there has been a growing interest in the role and function of females in innovation fields [44]. However, female participation in innovative activities is increasing globally. Many females have led innovation, entrepreneurship, and science and technology activities in a variety of companies. Although only 5.5% of commercial patent holders are female, female involvement in patent-intensive fields has been increasing [45].

In contemporary ICT companies, many innovative positions require relational as well as technical skills. The building and application of social capital, either actual or technology-based, are important to IB and KS. There are significant gender differences in the approaches used in the social networks, contacts, and alliances of colleagues to access knowledge and acquire new ideas, such that females tend to converse with others while males may experiment alone, when adapting to new technology [46].

The socialization process and gender role differences between genders can vary in terms of the construction and application of interpersonal trust. Interpersonal trust, as an element of social capital, is important for both male and female employees in sharing complete and true information and knowledge and taking risks to embrace IB [47]. However, males tend to construct and maintain an independent self-image and females consider others as parts of the whole self [48]. As compared to males, females tend to have smaller and lower-level professional networks and maintain high levels of interpersonal trust with their colleagues when participating in KS and organizational innovation [49]. For example, female scholars are more likely to cooperate with others to publish academic papers and males are more likely to publish alone, indicating that females may be more dependent on social capital to develop their innovations [50]. Thus, we expected that ITW, as a part of social capital, might have stronger impacts on KS and IB among female employees. Therefore, we developed the following two hypotheses:

**Hypothesis** **7** **(H7).** *ITW could have a stronger positive impact on KS for females than for males*.

**Hypothesis** **8** **(H8).** *ITW could have a stronger positive impact on IB for females than for males*.

**Figure 1:**
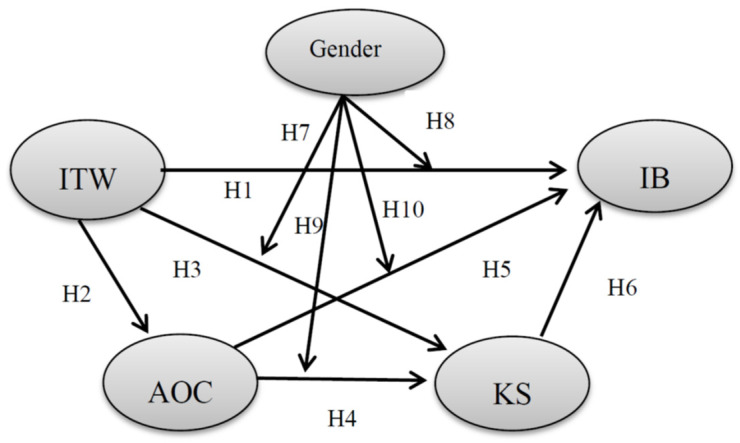
The hypothesized model. Notes: ITW = interpersonal trust in the workplace; AOC = affective organizational commitment; KS = knowledge sharing; IB = innovation behavior.

Previous research has suggested that males and females may use different criteria in their assessment of organizations. Males tended to have higher levels of organizational commitment than females, suggesting that males were more likely than females to hold jobs with commitment-enhancing features [51]. Females were faced with more barriers than males in gaining acceptance and identity in their organizations. Females usually worked in occupations not usually associated with innovation, while male employees had more expectations of gaining returns from their organizations, such as job security, career development, power and promotion, and future relationships [52]. Organizations might expect males to play key roles in most innovative activities in the natural sciences, technology and mathematics, and tacitly exclude females from these areas in the innovation policy [53]. When faced with the potential risks and costs of KS and employee innovation, male employees with higher AOC were more likely to voluntarily share knowledge and sacrifice self-interest to make innovation happen [54]. Prior research also demonstrated that male employees were more willing to share their knowledge with others and engage in innovative activities if they were certain that doing so was beneficial for their organizations [55]. Thus, higher AOC could lead to more KS and IB in males than in females. Therefore, we developed the following two hypotheses (the above hypotheses are shown in the research model in Figure 1):

**Hypothesis** **9** **(H9).** *AOC could have a stronger positive impact on KS for males than for females*.

**Hypothesis** **10** **(H10).** *AOC could have a stronger positive impact on IB for males than for females*.

## 3. Materials and Methods

### 3.1. Data Sources

This study analyzed data from an online survey conducted in 40 small and medium- sized ICT companies in Shanghai, China. Since we do not have the whole list of ICT companies in Shanghai, we selected the 40 companies using snow-ball sampling and then contacted the human resource managers at these companies to randomly select 20 respondents from each company. These human resource managers sent emails with links to the questionnaires to these anonymous respondents, using an online platform (https://www.wjx.cn/, accessed on 1 December 2021). The questionnaires include demographic questions and the questions addressing the constructs used in this study. A total of 686 respondents completed the online questionnaires. The response rate was 85.8%. Unfortunately, we did not have detailed information regarding the respondents who chose not to complete the questionnaires. Table 1 presents the characteristics of the sample.

### 3.2. Measurements

This study focused on the gender differences in the causal relationship between ITW and IB, while considering the moderating effects of AOC and KS. The measurements of these four factors were as follows.

IB was assessed by five items adopted from Zhou and George’s scale for employee innovation: suggesting new ways to achieve goals; often having new and innovative ideas; exhibiting creativity on the job when given the opportunity; developing adequate plans and schedules for the implementation of new ideas; and developing creative solutions to problems [37]. Each question was measured with the five-point Likert scale. IB did not only reflect the generation of innovative ideas and behaviors, but also reflected the promotion, development and implementation of innovative ideas, and finally promoted the transformation of innovative ideas into real products and technologies.

ITW was measured by a four-item scale adopted from McAllister’s interpersonal trust measure [56]. The interpersonal trust scale has been widely used to measure the trust among colleagues within an organization. The scale divides interpersonal trust into cognitive trust and affective trust. Cognitive trust is a rational trust based on a full understanding of others, while emotional trust reflects a combination of personal emotion with colleagues. As compared with cognitive trust, emotional trust is usually a more stable irrational trust. In this study, we modified the affective trust scale to measure the relationships between employees and their colleagues in the workplace. The four items were as follows: having a sharing relationship; having a sense of loss if one of us was transferred; colleagues responding constructively and caringly; and making considerable emotional investments in our working relationship.

AOC was measured by three items adopted from Allen and Meyer’s organizational commitment questionnaire: talking up this organization to my friends as a great organization to work for; my values and the organization’s values are very similar; and my organization really inspires the very best in me in the way of job performance [57]. This scale was proven valid for measuring organizational commitment in different organizations.

KS was measured by three items adopted from a Chinese version of the KS scale that was proven valid in the hi-tech companies: willing to actively tell others about my knowledge and experience; sharing new knowledge or new information with colleagues; and sharing notes, documents and data with colleagues [58]. The details of the items are shown in Table 2.

### 3.3. Analytical Technique

In this study, we evaluated the confirmatory factor analysis (CFA) models and structural equation models (SEM) with AMOS (IBM Company: Chicago, IL, USA). To compare the gender differences, we further applied the multi-group structural equation models. We analyzed the fits of these models to the data. We chose several goodness-of-fit indices for the analyses: chi-squared, comparative fit index (CFI), goodness-of fit-index (GFI) and the root mean square error of approximation (RMSEA). We tested several multi-group models with different restrictions and achieved the best model with possible restrictions on causal relationships [59].

## 4. Results

### 4.1. Descriptive Results

Table 1 reported the characteristics of the participants between gender groups. Approximately 48% of respondents from these 40 ICT companies were male (n = 332), as comparison to females at 52% (n = 356). Males tended to have higher levels of IB, KS and AOC than females, although the differences are not very significant. The means of four items of ITW were slightly higher for females than for males. These indicated that the gender differences in ITW were not very significant.

### 4.2. Results from Confirmatory Factor Analysis Models

We first evaluated the CFA models. As shown in Table 2, all factor loadings were greater than 0.5, indicating that the measurements of these four latent variables had high validity [59]. The four latent variables, namely IB, ITW, KS and AOC, were highly correlated. The fitting indices of the model were: CFI = 0.965, GFI = 0.924, and RMSEA = 0.067. According to the modification indices, we added a correlation between the error items of I-2 and I-4. The model fitness increased as follows: CFI = 0.973, GFI = 0.934, and RMSEA = 0.059. The revised CFA model had a good fit to the data.

The correlations among latent variables in the CFA model are shown in Table 3. All of the correlations were statistically significant. Their standardized coefficients ranged from 0.56 to 0.786, indicating strong ties among these latent factors.

### 4.3. Results from the Structural Equation Models

We conducted a structural equation model after the implementation of the CFA models for the purpose of examining the research model and the first six hypotheses. The fitting indices of the model were acceptable: CFI = 0.983, GFI = 0.961, and RMSEA = 0.046.

Table 4 reported the standardized causal relationships among these latent factors in this structural equation model. First of all, the statistical results showed that the structural relationships among the latent factors were significant and strong, supporting hypotheses H1–H6. ITW had direct impacts on AOC, KS and IB. The standardized coefficient for the path from ITW to AOC reached 0.69, while those to KS and IB were 0.34 and 0.15, respectively. AOC had a strong effect on KS (=0.41) but only a moderate effect on IB (=0.16). The standardized coefficient for the path from KS to IB was 0.59, indicating the importance of KS for IB. The standardized indirect effect of ITW on IB reached 0.481, whereas its direct effect was only 0.147. These results revealed that ITW had medicating effects through AOC and KS to predict IB. Similarly, AOC also had a significant indirect effect through KS to IB.

### 4.4. Results from Multi-Group SEMs

To test the moderate effect of gender on the structural relationships, we divided the sample into two gender sub-samples, and then applied multi-group SEMs. The unrestricted multi-group model had good fitness: CFI = 0.979, GFI = 0.941, and RMSEA = 0.037. In this model, as reported in Table 5, ITW and AOC had no significant effects on IB in the male group, indicating that KS could fully mediate the impacts of ITW and AOC on IB. However, all the coefficients of the causal relationships were significant in the female group. These results illustrated that for female employees, both the direct and indirect effects of ITW and AOC on IB were significant.

We further tested four models with different restrictions. Table 6 reports the estimation results of multi-group SEMs with the same unrestricted and restricted measurement as well as structural weights. There were significant differences in the chi-squared values between multi-group SEMs with the same measurement, structural weights and the unrestricted weights. We then released the restriction of the factor-loading from the latent factor of KS to one of its items. The revised model showed no difference in the chi-squared values, as compared to the unrestricted model.

Based on the revised model, we added restrictions on the structural weights one by one. The chi-squared test showed that the restrictions of the same structural weights from ITW to IB, from ITW to KS and from AOC to KS significantly increased the chi-square results, indicating important gender differences in these structural relationships. Adding the last three restrictions to the model did not significantly change the model’s fit.

Table 7 shows the structural relationships with the same three weight restrictions between male and female sub-samples. For both male and female employees, ITW increased AOC and KS. AOC encouraged IB. However, the impacts of ITW on both IB and KS were much stronger for females than for males. AOC significantly increased the KS for males than for females. In other words, the mediation effect of AOC between ITW and IB is stronger in the male sub-sample.

## 5. Discussion

The objective of this study was to provide insights into the gender differences involved in ITW on IB, while considering AOC and KS as mediators. We applied CFA and multi-group SEMs and surveyed 688 respondents from 40 ICT companies in Shanghai, China. The results contributed to the literature regarding gender gaps as well as the causal relationship between ITW and IB.

### 5.1. The Structural Relationships among ITW, AOC, KS and IB

The results of the structural relationships based on the total sample confirmed the positive effect of ITW on IB, supporting the hypothesis H1. ITW was helpful for creating an inclusive innovation culture, improving employees’ enthusiasm for innovation, reducing opportunistic behavior, and providing emotional safety and psychological support for employees to stimulate their IBs in ICT companies. The results showed that increased ITW also encouraged more risk-taking behaviors and then improved the innovation performance of the ICT companies, consistent with the previous studies [6,7].

Previous studies demonstrated that ITW not only promotes employees’ creativity, but also increases AOC and KS [11,18]. This study confirmed that both AOC and KS played important mediating roles between ITW and IB, supporting hypotheses H2–H6. Employees with higher ITW usually had stronger AOC and felt more supported. They were also more likely to share knowledge and experiences with their colleagues in the workplace. These results provided the evidence to highlight both ITW and AOC in innovation management and knowledge management.

### 5.2. Gender Differences in the Relationship between ITW and IB

This study revealed that the direct impact of ITW on IB was stronger for females than for males, supporting the hypothesis H8. Similarly, female employees were more likely to share knowledge with others if they trusted their colleagues, supporting the hypothesis H7. It verified that female employees were more reliant on social ties and preferred working with co-workers’ supports [46]. Female employees believed that IB was more likely to be effective with the helping hands of coworkers. Thus, ITW was more related to KS and IB among female employees, as compared to males [43].

In contrast, KS was more related to AOC among male employees than females, providing evidence for the hypothesis H9. Previous studies on the relationship between AOC and KS hardly addressed the issue of the gender difference. This study revealed that stronger AOC encouraged male employees to share knowledge, even though it might be costly and risky. Due to the consistent correlations of IB with KS and AOC across genders, the indirect effect of AOC on IB via KS was stronger for male employees. The reasons why the AOC of male employees is highly correlated with KS could be that they had been socialized to favor loyalty and righteousness to their organizations. Similarly, the reason why female KS was less correlated with AOC could be that they had been socialized to respect cooperation with their surroundings [60].

This study further revealed that the influence of AOC on IB was almost unchangeable across genders, providing no evidence for the hypothesis H10. In addition, the influence of ITW on AOC and the influences of AOC and KS on IB are similar across genders. These findings confirmed that the path from ITW via AOC to IB was important for both male and female employees in ICT companies.

### 5.3. Theoretical Implication

Although social determinants of IB in the workplace have been extensively studied, not so many studies have addressed the gender differences in its relationships with ITW, AOC and KS. Previous research has revealed the mediating effect of KS between ITW and IB [4,11]. This study elucidated the differences in the ITW–IB relationship between male and female employees, trying to contribute to the literature on gender gaps. ITW affected male IB and KS more, whereas AOC affected female KS more than male KS. These empirical results support our hypotheses derived from the previous discussion. To our knowledge, this study is the first to explain the difference in the ITW–IB relationship between genders in ICT companies. We could explain these gender differences by relying on social exchange and social capital theory, which highlight the effects of social support and social trust on female IB [42]. Compared to male employees, female employees were more likely to apply social capital, such as trusted networks, to access new knowledge and to integrate it with their own personal knowledge to make innovation [44]. Female employees with good relationships with their colleagues were more confident that their IBs and KSs could result in positive feedbacks. They were more dependent on the promotive context for innovation given by their coworkers or their team members. Female employees might be more likely to receive supports, encouragement and positive feedback from their colleagues. Both male and female coworkers were usually willing to assist a female employee faced with technical obstacles in an ICT company and as a result, she would be less concerned about the negative consequences of IB and KS [48]. Female employees would be less innovative and share less knowledge if they distrusted their co-workers or felt less supportive in the workplace.

By distinguishing gender differences alone in the path from AOC to KS, the results provided the next step to advance the previous research in the field of knowledge management. This study extended the research model for the relationship between AOC and KS. Although the existing literature suggested that both AOC and ITW were important for KS [11,37], this present study indicated that AOC was much more important for KS among male employees than females. Why was such a mediating effect stronger for men? One explanation could be the gender differences in technology acceptance and usage behaviors. Male employees were more willing to share their knowledge with others if they considered these behaviors useful for their organizations [55].

### 5.4. Management Inspiration

The main contributions of this study were related to gender differences in the influence of ITW on IB and the mediating roles of AOC and KS in ICT companies. The results highlighted the relevance of ITW, AOC and KS in the promotion of IB. Therefore, the managers of the ICT companies that encourage more IBs should increase KS through the development of an organizational environment based on ITW and AOC. Such results showed the managers the importance of encouraging a trustful environment for female employees in terms of KS and IB. In order to nurture a trust-based organizational environment, managers should design human resource practices related to ITW, such as developing team competency, providing group rewards, and establishing workload sharing [61]. Such activities can be more effective to enhance female ITW and lead to more KS and IB.

This study also addressed the importance of AOC for KS among male employees in ICT companies. KS and AOC were characteristics of the organizational culture and support organizational innovation. This study provided evidence regarding supporting KS to male employees. Hi-tech companies can benefit from stimulating KS among male employees as a result of their AOC. Managers should design team-building activities and other managerial practices related to organizational commitment, such as mentoring programs and employee orientation processes, according to the needs of male employees [62].

### 5.5. Research Limitations

Some limitations of this study were worth noting and suggested possible avenues for further research. First, the sample adopted in this study was limited to 688 respondents from 40 Chinese ICT companies and thus caused some concerns over the generalizability of the results. There was also an intention to test the gender differences in other organizations and cultural contexts. Second, this study used cross-sectional data that limited the conclusions about causality. Thirdly, future studies could also improve the explanatory power of the model proposed by adding more variables that could more comprehensively explain the mediating mechanisms through which organizational environments shaped KS and innovative behaviors. Moreover, expanding the gender differences in the influencing mechanisms of different types of organizational commitment might also theoretically further relevant studies in the future.

## 6. Conclusions

This study assessed the role of gender differences in the effect of interpersonal trust on employee innovation and the mediating roles of organizational commitment and KS. The results from structural equation models showed that ITW had significant impacts on AOC, KS and IB. AOC and KS mediated the effect of ITW on IB. Furthermore, the direct impact of ITW on IB was significantly higher for females than for males, whereas male AOC increased KS. In addition, there were no significant gender differences on the effect of ITW on AOC and on the effect of KS on IB. The results confirmed that ITW was more important for female IB, and AOC was more important for male KS. This study provided a better understanding for managers regarding gender differences and encouraging employee innovation.

## Figures and Tables

**Table 1:** Demographic characteristics of the sample.

	Frequency	(%)
Gender:		
Female	332	48.26
Male	356	51.74
Age:		
Below 25	139	20.2
25–29	297	43.17
30–34	145	21.08
35–39	74	10.76
Above 40	33	4.8
Education degree:		
Below bachelor’s degree	116	16.86
Bachelor’s degree	467	67.88
Master’s degree and above	105	15.27
Job position:		
Manager	151	21.95
Marketing and service	173	25.15
Engineer	144	20.93
Other	220	31.98

**Table 2:** Factor loadings of the confirmatory factor analysis model and means between genders.

Item	Factor Loading	Mean	
		Female (n = 356)	Male (n = 332)
IB:			
B-1: I suggest new ways to achieve goals.	0.80	4.146	4.205
B-2: I often have new and innovative ideas	0.79	3.817	3.997
B-3: I exhibit creativity on the job when given the opportunity to	0.87	4.053	4.175
B-4: I develop adequate plans and schedules for the implementation of new ideas	0.85	4.059	4.148
B-5: I develop creative solutions to problems	0.86	3.958	4.145
ITW:			
I-1: We have a sharing relationship. We can both freely share our ideas, feelings, and hopes	0.86	4.430	4.380
I-2: We would both feel a sense of loss if one of us was transferred and we could no longer work together	0.55	3.834	3.765
I-3: If I shared my problems with my colleagues, I know they would respond constructively and caringly	0.87	4.228	4.220
I-4: I would have to say that we have both made considerable emotional investments in our working relationship	0.71	3.966	3.916
AOC:			
A-1: I talk up this organization to my friends as a great organization to work for	0.84	4.051	4.108
A-2: I find that my values and the organization’s values are very similar	0.90	4.065	4.099
A-3: My organization really inspires the very best in me in the way of job performance	0.84	4.104	4.120
KS:			
K-1: I am willing to tell others about my knowledge and experience actively	0.72	4.118	4.205
K-2: When I have new knowledge or new information, I will share it with my colleagues	0.76	3.921	4.069
K-3: When my colleagues are in need of notes, documents and data, I am willing to share with them	0.62	4.228	4.322

Notes: ITW = interpersonal trust in the workplace; AOC = affective organizational commitment; KS = knowledge sharing; IB = innovation behavior.

**Table 3:** Correlations among the latent variables in the revised CFA model.

Correlation			Coef.	S.E.	C.R.	St. Coef.
ITW	<--->	EI	0.235	0.031	7.614	0.560
ITW	<--->	AOC	0.388	0.044	8.737	0.694
ITW	<--->	KS	0.279	0.037	7.589	0.568
AOC	<--->	EI	0.291	0.036	8.166	0.636
AOC	<--->	KS	0.359	0.043	8.282	0.670
KS	<--->	EI	0.316	0.035	9.146	0.786

Note: Coef. = coefficient; S.E. = standard error; C.R. = critical ratios; St. Coef. = standardized coefficient. “<--->” refers to the correlation between two lateent variables.

**Table 4:** Standardized coefficients of the structural effects on IB.

		ITW	AOC	KS
	AOC	0.689	-	-
Total effects	KS	0.624	0.412	-
	EI	0.628	0.404	0.594
	AOC	0.689	-	-
Direct effects	KS	0.340	0.412	-
	EI	0.147	0.160	0.594
Indirect effects	KS	0.284	-	-
	EI	0.481	0.245	-

**Table 5:** Results from the unrestricted multi-group structural equation models.

			Coef.	S.E.	C.R.	*p*
Male group:						
AOC	<---	ITW	0.757	0.065	11.588	***
KS	<---	ITW	0.190	0.074	2.559	0.010
KS	<---	AOC	0.468	0.071	6.570	***
EI	<---	ITW	0.086	0.052	1.639	0.101
EI	<---	KS	0.541	0.061	8.828	***
EI	<---	AOC	0.104	0.056	1.846	0.065
Female group:						
AOC	<---	ITW	0.881	0.073	12.050	***
KS	<---	ITW	0.497	0.074	6.703	***
KS	<---	AOC	0.190	0.054	3.553	***
EI	<---	ITW	0.245	0.064	3.816	***
EI	<---	KS	0.449	0.062	7.295	***
EI	<---	AOC	0.124	0.042	2.973	0.003

Note: Coef. = coefficient; S.E. = standard error; C.R. = critical ratios; St. Coef. = standardized coefficient. “<---” refers to the causal relationship from the variable on the right to the variable on the left. *** *p* < 0.001.

**Table 6:** Comparison of the fit of the multi-group structural equation models.

Model	CMIN	DF	CMIN/DF	CFI	RMSEA	LO 90	HI 90
1. Unlimited	952.460	476	2.001	0.959	0.038	0.035	0.042
2. Same co-variances	962.749	497	1.937	0.960	0.037	0.033	0.040
3. Same intercepts	1005.820	521	1.931	0.958	0.037	0.033	0.040
4. Same structural relationships	1022.162	524	1.951	0.957	0.037	0.034	0.041
5. One restriction Released	1006.203	523	1.924	0.958	0.037	0.033	0.040

Note: CMIN = chi-squared value; DF = degree of freedom; CMIN/DF = discrepancy divided by the degree of freedom; CFI = comparative fit index; RMSEA = root mean square error of approximation; LO 90 = lower boundary of a 90% confidence interval of the RMSEA; HI 90 = higher boundary of a 90% confidence interval of the RMSEA.

**Table 7:** Structural relationships with the same weight restrictions between gender groups.

			Coef.	S.E.	C.R.	*p*
Male group:						
AOC	<---	ITW	0.820	0.049	16.796	***
KS	<---	ITW	0.176	0.073	2.405	0.016
KS	<---	AOC	0.444	0.066	6.721	***
EI	<---	ITW	0.093	0.044	2.098	0.036
EI	<---	KS	0.500	0.043	11.743	***
EI	<---	AOC	0.119	0.034	3.528	***
Female group:						
AOC	<---	ITW	0.820	0.049	16.796	***
KS	<---	ITW	0.512	0.072	7.069	***
KS	<---	AOC	0.208	0.056	3.739	***
EI	<---	ITW	0.214	0.050	4.321	***
EI	<---	KS	0.500	0.043	11.743	***
EI	<---	AOC	0.119	0.034	3.528	***

Note: Coef. = coefficient; S.E. = standard error; C.R. = critical ratios; St. Coef. = standardized coefficient. “<---” refers to the causal relationship from the variable on the right to the variable on the left. *** *p* < 0.001.

## Data Availability

Data collected and analyzed during the study are available upon reasonable request.
